# External Esophagotomy for Impacted Foreign Body in the Esophagus, Located by X-Ray

**Published:** 1899-02

**Authors:** 


EXTERNAL ESOPHAGOTOMY FOR IMPACTED FOREIGN BODY IN THE ESOPHAGUS, LOCATED BY X-RAY.—By Dr. J. McCoy (*N. Y. Med. News*, 1898, LXXIII., No. 23, p. 709).—The author reports a case of esophagotomy upon a boy who had swallowed a tin whistle, which was subsequently located by X-ray in the median line of the neck, about an inch above the sternal notch.

In the light of this case the author draws the following conclusions:

1. A foreign body impacted in the esophagus for more than twelve hours, particularly if it be known to have edges liable to inflict injury upon the tissues if traction be made upon it, should be removed at once by external incision.

2. The prolonged and continued used of the various esophageal instruments should be condemned, as the tissues are usually contused and lacerated by their use and their vitality and resisting power lowered.

3. When the tissues have not been lacerated and the impaction has not been of long duration, the tissues appearing healthy, the esophagus should be closed with fine catgut and the external wound closed completely.

4. It is better to give small portions of sterilized water at an early hour after the operation, in order that the esophagus may be kept as clean as possible.

Feeding by tube or per rectum is usually uncalled for. At the end of twenty-four hours liquid nourishment may be given, and after each feeding a few sips of sterilized water should be administered, so that the esophagus may be again cleansed.

